# In Vitro Tests for a Rapid Evaluation of Antidiabetic Potential of Plant Species Containing Caffeic Acid Derivatives: A Validation by Two Well-Known Antidiabetic Plants, *Ocimum gratissimum* L. Leaf and *Musanga cecropioides* R. Br. ex Tedlie (Mu) Stem Bark

**DOI:** 10.3390/molecules26185566

**Published:** 2021-09-13

**Authors:** Abdulmomem Awwad, Patrick Poucheret, Yanis A. Idres, Damien S. T. Tshibangu, Adrien Servent, Karine Ferrare, Françoise Lazennec, Luc P. R. Bidel, Guillaume Cazals, Didier Tousch

**Affiliations:** 1 Qualisud, University Montpellier, CIRAD, Institut Agro, Avignon Université, University de La Réunion, 34090 Montpellier, France; abd.awad.fr@gmail.com (A.A.); yanidres@gmail.com (Y.A.I.); adrien.servent@cirad.fr (A.S.); karine.ferrare@umontpellier.fr (K.F.); francoise.lazennec@umontpellier.fr (F.L.); didier.tousch@umontpellier.fr (D.T.); 2 Laboratory of Natural Substances and Medicinal Chemistry, Faculty of Sciences, University of Kinshasa, Kinshasa 190 Kinshasa XI, Democratic Republic of the Congo; tshibangud@gmail.com; 3 INRA, UMR AGAP, CIRAD, SupAgro, 2 Place Pierre Viala, 34060 Montpellier, France; luc.bidel@umontpellier.fr; 4 Guillaume Cazals, University Montpellier, IBMM UMR5247, CNRS, ENSCM, Place Eugène Bataillon, CEDEX 5, 34095 Montpellier, France; guillaume.cazals@umontpellier.fr

**Keywords:** *Ocimum gratissimum*, *Musanga cecropioides*, in vitro antidiabetic, insulin secretion, glucose uptake, protection cells against H_2_O_2_ oxidative stress, caffeoyl derivatives

## Abstract

Plant bioactive extracts represent a major resource for identifying drugs and adjuvant therapy for type 2 diabetes. To promote early screening of plants’ antidiabetic potential, we designed a four in vitro tests strategy to anticipate in vivo bioactivity. Two antidiabetic plants were studied: *Ocimum gratissimum* L. (Oc) leaf extract and *Musanga cecropoides* R. Br. ex Tedlie (Mu) stem bark extract. Chemical compositions were analyzed by LCMS and HPLC. Antidiabetic properties were measured based on (1) INS-1 cells for insulin secretion, (2) L6 myoblast cells for insulin sensitization (Glut-4 translocation), (3) L6 myoblast cells for protection against hydrogen peroxide (H_2_O_2_) oxidative stress (cell mortality), and (4) liver microsomial fraction for glucose-6-phosphastase activity (G6P). Oc extract increased insulin secretion and insulin sensitivity, whereas it decreased oxidative stress-induced cell mortality and G6P activity. Mu extract decreased insulin secretion and had no effect on insulin sensitivity or G6P activity, but it increased oxidative stress-induced cell mortality. Results were compared with NCRAE, an antidiabetic plant extract used as reference, previously characterized and reported with increased insulin secretion and insulin sensitivity, protection against oxidative stress, and decreased G6P activity. The proposed set of four in vitro tests combined with chemical analysis provided insight into the interest in rapid early screening of plant extract antidiabetic potential to anticipate pharmaco-toxicological in vivo effects.

## 1. Introduction

Type 2 diabetes (T2D) is a metabolic disorder characterized by uncontrolled chronically high glucose levels. It is now admitted that chronic oxidative stress and low grade chronic inflammation are associated with the insulin resistance of insulin-sensitive tissues, e.g., skeletal muscles, liver, and adipose tissue [1]. Under this environmental imbalance, the β cells are subjected to a glucose toxicity effect as well as to oxidative stress, inflammation stress, endoplasmic reticulum stress, and amyloid stress, leading to abnormal insulin secretion and cell death in the long term [2,3].

To treat T2D, oral pharmacological antidiabetic allopathic drugs are prescribed in order to reduce hyperglycemia. These drugs include insulin secretagogue agents (sulfonylureas, glinides), insulin-sensitizing agents (metformin), and glucose absorption inhibitors such as α-glucosidase inhibitors (acarbose, miglitol). Although these drug molecules are used effectively, none of them are simultaneously targeting oxidative stress/chronic inflammation and insulin resistance.

In this context, plant extracts represent a potential adjuvant therapy as food supplements or health foods [4]. The hydroxycinnamic acids, including caffeic derivatives such as chlorogenic and chicoric acids, possess antidiabetic effects. They combine anti-inflammatory activity with protection against oxidative stress and cytotoxic injuries, but also provide insulin-sensitizing properties to tissues and/or an insulin-secreting effect [5,6,7,8,9,10,11]. In a previous work, we studied a caffeic derivatives extract from *Chicorium intybus* L. root (NCRAE) containing a high CRA (chicoric acid) content. It was investigated using four in vitro tests. We showed that NCRAE (at a concentration range of 10–100 µg·mL^−1^) was able to increase insulin secretion of INS-1 cells, increase the insulin sensitization of L6 myoblast cells [8], protect the L6 cells against an oxidative H_2_O_2_ stress, and decrease the glucose-6-phosphatase (G6Pase) activity in rat liver microsomes [10]. The NCRAE in vitro effects were correlated with an in vivo antidiabetic effect [11].

Based on our NCRAE results, we intended to study the pertinence of a set of four in vitro tests, using a single dose of 50 µg·mL^−1^ concentration, in order to assign or not an antidiabetic potential to a plant extract. Thus, to avoid in vivo test burden, this one shot strategy could allow a greater efficiency in screening the antidiabetic potential of a large collection of extracts.

Among the candidate plants known for their antidiabetic effects and caffeic derivatives contents, we used *Ocimum gratissimum* L. (Lamiaceae family) and *Musanga cecropioides* R. Br. ex Tedlie (Cecropiaceae family). In vivo studies demonstrated that Oc leaf extracts possess both in vitro and in vivo hypoglycemic properties [12,13,14,15,16,17,18,19]. Recently, *Musanga cecropioides* (Mu) leaves or stem bark extracts have shown an antidiabetic potential [20,21,22,23,24]. A Mu leaf extract exhibited an anti-inflammatory effect in a specific animal model [25]. The chemical analysis of *Ocimum gratissimum* content demonstrated the presence of some caffeic derivatives with caffeic, caftaric, and chicoric acids being the most abundant [19,26]. In *Musanga cecropioides*, the presence of chlorogenic acid was also revealed [27,28].

Our approach was to investigate the in vitro effects of extracts from *Ocimum gratissimum* and *Musanga cecropioides* in comparison with previous results obtained with *Cichorium intybus* extract (NCRAE). After chemical analysis of the caffeic derivatives content was realized, the in vitro investigations were carried out: (i) insulin secretion test on INS-1 cells, (ii) glucose uptake evaluation of L6 muscle cell, (iii) cell protection test against H_2_O_2_ oxidative stress in L6 cells, (iv) glucose 6-phosphatase (G6Pase) activity in hepatic microsomal fraction.

## 2. Results

**Figure 1:**
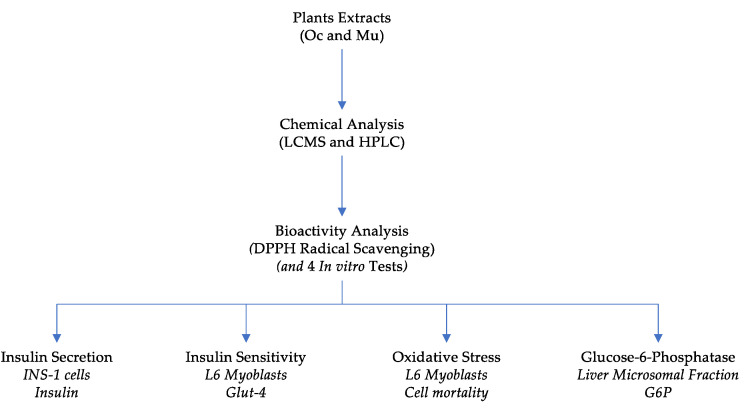
Workflow diagram.

Results were obtained following the workflow diagram presented hereafter (Figure 1).

### 2.1. Chemical Analysis of Oc and Mu

#### 2.1.1. LC-MS Analysis 

Oc extract had three major peaks, corresponding to chicoric acid (P30), rosmarinic acid (P30^1^), and tri-caffeoylquinic acid isomer (P32), in accordance with previous publications [19,26]. In addition, this extract had three apigenin glycoside derivatives (P12, P14, P16)) with the shared fragment (*m*/*z* [M + H]^+^) = 409 [M + H]^+^) and minor peaks corresponding to flavones derivatives luteolin (P6) and apigenin glycosides, as reported [29]. Finally, the extract was rich in one dihydroxybenzoic acid—(P3), as expected [30]. The extraction and purification process strongly depleted the more hydrophilic compounds of leaves of *Ocimum basilicum* previously described (gallic acid, gallic acid methyl ester, gallic acid ethyl ester, 3,4-dihydroxybenzoic acid, 4-hydroxybenzoic acid, 4-hydroxybenzaldehyde, gentisic acid, vanillic acid, caffeic acid). Some additional hydrophobic compounds previously described were also found at trace level (*p*-coumaric acid, ferulic acid, circiliol, cirsimarin, cirsilineol, acacetin, eryodictyiol-7-O-glucoside, eugenol) [30].

The chemical analysis of Mu stem bark extract was in accordance with the literature [27,28,31]. In the minor peaks, four hydroxycinnamic acids were identified (P10, P23, P25 to P32), most notably chlorogenic acid (P10) and caffeoyl tartaric acid or caftaric acid (P23). Three dihydroxybenzoic acids were found (P1, P2, P3, P4) with the protocatechuic acid (P1) being the most abundant, as previously described [28]. The 2,3-dihydroxybenzaldehyde (P5) was also found (at 5.8 min, 8.7 min, and 17.5 min). The Mu extract contained two major peaks, exhibiting absorbance spectrum of apigenin (P12 and P15). The first (maxima at 269.9 and 335.9 nm, with *m*/*z* = 593 [M − H]^−^, had fragments ions *m*/*z* = 431, and 269 [M − H]^−^) was identified as an apigenin di-hexoside (apigenin 6,8-di-C-glucoside) (P12). The second (maxima 270.9 and 337.9 nm, with *m*/*z* = 563 [M − H]^−^, and fragments ions 431 and 269) was tentatively assigned to an apigenin-C-pentosyl-C-hexoside (P15). Eight minor flavonoidic pics shared a typical absorbance spectrum of apigenin (P13, P15, P18 to P22, P24). In the case of P21 and P22, the two fragments *m*/*z* = 341 and 311 corresponded to fragments [M − H − 120−30]^−^ and [M − H − 120]^−^, respectively and were characteristic of the cleavage of one glycosyl moiety. Their fragment *m*/*z* = 269 [M – H − 164]^−^ corresponded to the aglycone [Apigenin-H]^−^. They corresponded to apigenin-8-C-glucoside (vitexin, P21) and apigenin-6-C-glucoside (isovitexin, P22), already found in stem bark of *Musanga* [28]. In positive mode, these compounds formed fragments shared by both vitexin and isovitexin (*m*/*z* = 121, 259, 283, 284, 313, 314 [M + H]^+^), and two major fragments *m*/*z* = 337 and 415 [M+H]^+^, which are described as solely specific to isovitexin fragmentation and corresponding to [M + H − 18]^+^ and [M + H − 96]^+^ in accordance with a previous report [31] (Table 1). 

#### 2.1.2. HPLC Analysis

**Figure 2:**
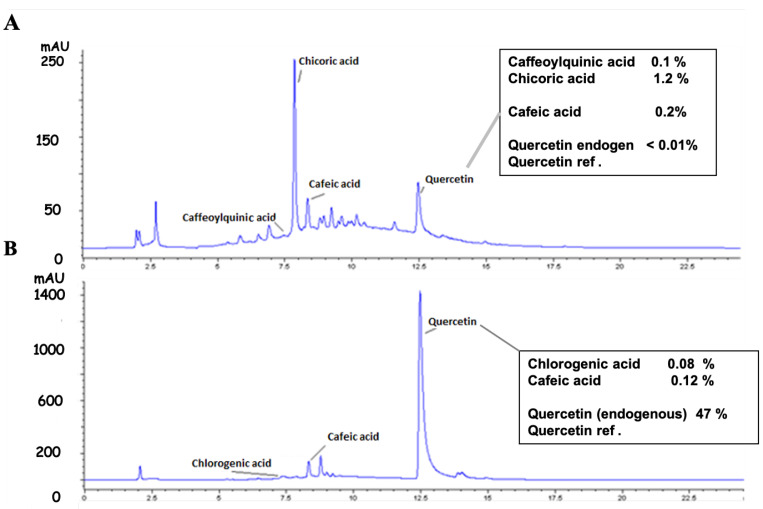
Quantification of caffeoyl derivatives in Oc and Mu leaves extracts by HPLC. (**A**) chromatogram obtained with *Ocimum gratissimum* L. extract (Oc). (**B**) chromatogram obtained with *Musanga cecropoides* R. Br. ex Tedlie (Mu). A standard solution containing chlorogenic acid, caffeic acid, and chicoric acid at 10 µM each were used as peak area references. A 5 µM external quercetin marker was subsequently added to Oc and Mu extracts to normalize the data analysis. The high uptick found in Mu represents both quercetin (the endogene part and the reference added) and probably the apigenin contents in Mu extract.

HPLC analysis allowed evaluation of the percentage of caffeic derivatives in the two extracts. We found in Oc (Figure 2A) and Mu (Figure 2B), respectively: 0.21% and 0.08% of chlorogenic acid, 0.2% and 0.12% of caffeic acid, 0.01% and 47% of apigenin or derivatives. Oc contained 1.2% of chicoric acid. No chicoric acid was detected in Mu even though caffeoyl tartaric acid was detected by LC-MS (Figure 2). 

#### 2.1.3. DPPH Free-Radical Scavenging Capacities of Oc and Mu Extracts

The DPPH free-radical scavenging capacities, expressed as QE·mg^−1^ for Oc and Mu extracts, were respectively 615 ± 15 nmoles·mg^−1^ and 1900 ± 15 nmoles·mg^−1^. In comparison, the Quercetin IC_50_ was 20 µg·mL^−1^, whereas we obtained an IC_50_ at 30.9 ± 1.5 µg·mL^−1^ and 9.97 ± 0.9 µg·mL^−1^ for Oc and Mu extracts, respectively.

### 2.2. Oc and Mu In Vitro Effects

#### 2.2.1. Insulin Secretion Evaluations

**Figure 3:**
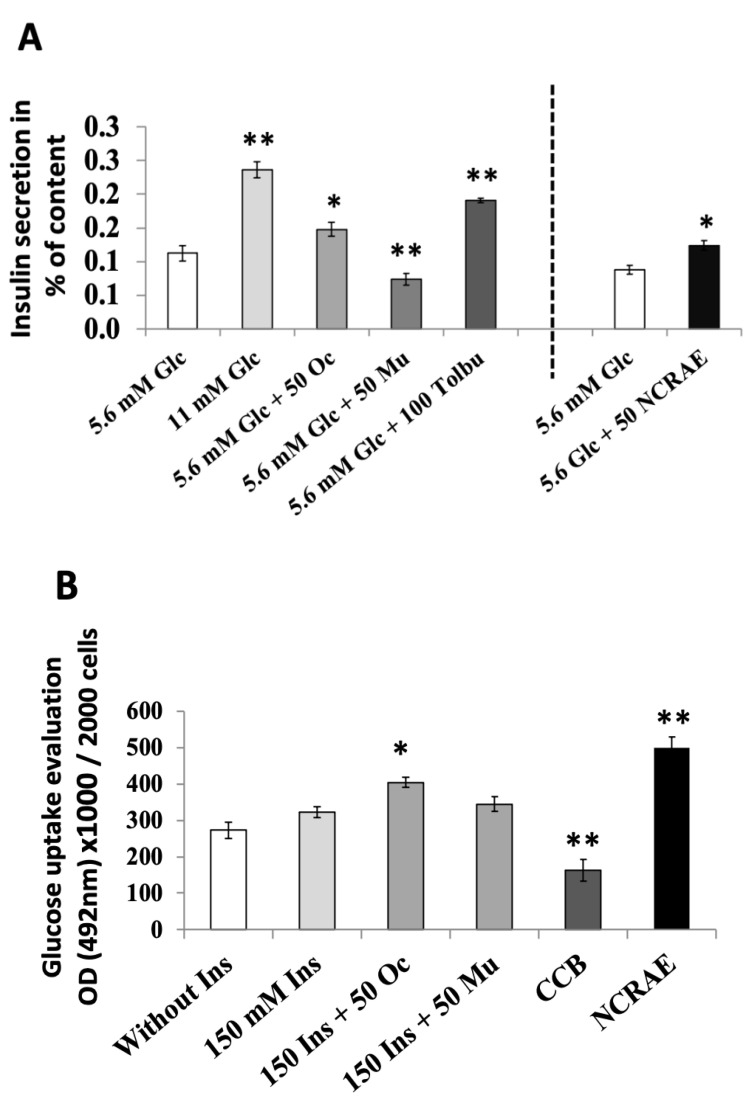
Insulin release and glucose uptake effects of Oc extract or Mu extract. (**A**) Insulin release of INS1 β cells in the presence of 5.6 mM glucose, with or without 50 µg·mL^−1^ of extracts (Oc and Mu). Comparison with NCRAE (reference extract) and tolbutamide as control. Values are the means (±SD) of three independent experiments (* *p* < 0.05 and ** *p* < 0.01). (**B**) Glucose uptake evaluation of L6 muscles cells in the presence of 150 nM insulin, with or without 50 µg·mL^−1^ of extracts. Comparison with NCRAE and cytochalasin B (CCB) as positive control. Values are the means (±SD) of ten independent experiments. (* *p* < 0.05 and ** *p* < 0.01.).

Insulin release by INS-1 β cells displayed a clear and normal glucose dependency from 5.6 to 11 mM glucose concentration. In the presence of 5.6 mM glucose, Oc extract (50 µg·mL^−1^) induced a significant increase in insulin secretion (*p* < 0.05). In contrast, Mu extract (50 µg·mL^−1^) generated a drop in insulin secretion (*p* < 0.01). Positive control, tolbutamide (100 µM), provoked a clear increase in insulin release (*p* < 0.01) as expected. NCRAE (50 µg·mL^−1^) induced an equivalent insulin secretion when compared with Oc extract (Figure 3A).

#### 2.2.2. Insulin Sensitizing Evaluations

As expected, in the presence of 150 nM insulin, GLUT4 quantification on the membrane cell of L6 cells was higher than controls. The addition of Oc extract at a concentration of 50 µg·mL^−1^ significantly increased GLUT4 transporters’ presence in the membrane (*p* < 0.05). At the same concentration, Mu extract did not significantly modify GLUT4 quantification. As expected, the reference inhibitor, cytochalasin (CCB), effectively reduced the translocation of GLUT4 to the membrane (*p* < 0.01). NCRAE induced the highest increase in GLUT4 transporters toward the membrane (*p* < 0.01, Figure 3B). 

#### 2.2.3. Hepatic Glucose 6-Phosphatase Activity

**Figure 4:**
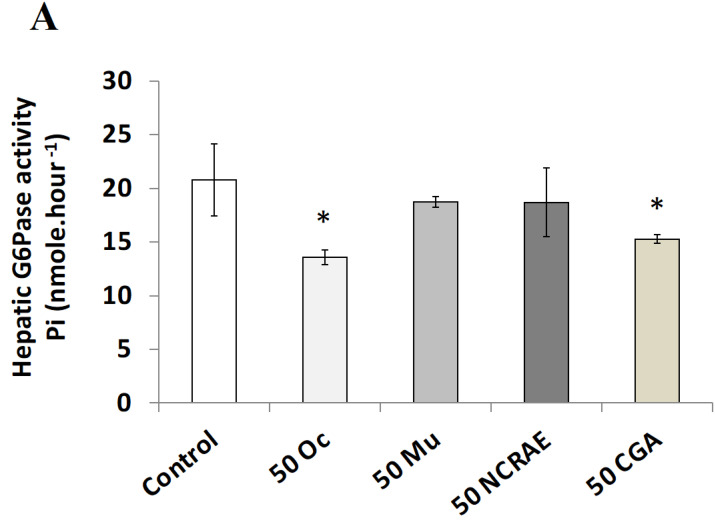

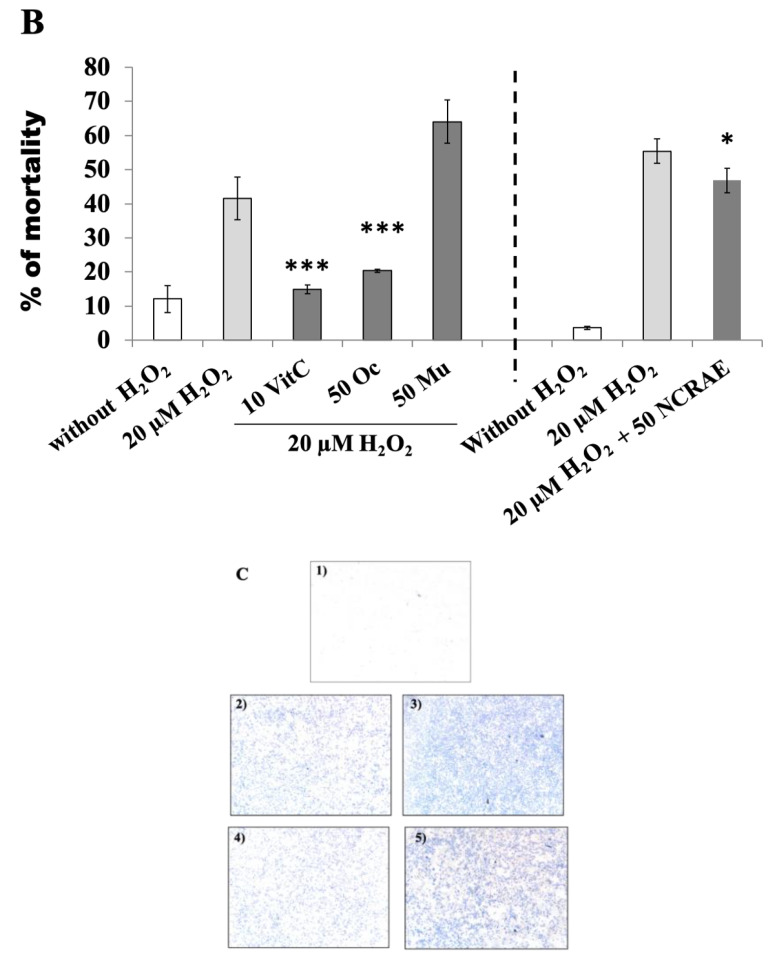
Modulation of the glucose 6-phosphatase activity and H_2_O_2_ oxidative stress effects of Oc extract or Mu extract (50 µg·mL^−1^). (**A**) Glucose 6-phosphatase activity on hepatic microsomes in presence of Oc and Mu extracts. Comparison with NCRAE as reference extract and chlorogenic acid (CGA) as positive control. Values are the means (±SD) of three independent experiments (* *p* < 0.05). (**B**) Percentage of mortality induced in L6 muscle cells by H_2_O_2_ oxidative stress. Comparison with NCRAE (reference extract) at 50 µg·mL^−1^ and vitamin C (Vit C) as positive control. Values are the means (±SD) of six independent tests (* *p* < 0.05, and *** *p* < 0.001). (**C**) Images of L6 cells mortality after H_2_O_2_ oxidative stress exposure, objectivized by trypan-blue staining. (1) Control without H_2_O_2_; (2) with 20 µM H_2_O_2_; (3) with 60 µM H_2_O_2_; (4) Oc pretreatment and 20 µM H_2_O_2_; (5) Mu pretreatment and 20 µM H_2_O_2_.

G6Pase activity was measured on rat microsomal hepatic fraction. Oc extract (50 µg·mL^−1^) induced a clear inhibitory effect (*p* < 0.05). At the same concentration, Mu extract did not significantly modify this activity, despite the significant CGA content, suggesting that the potential presence of other(s) molecule(s) counteracting CGA effects, as hypothesized in the Discussion. Likewise, NCRAE did not influence hepatic G6Pase, whereas chlorogenic acid (CGA) at 50 µg·mL^−1^ induced a significant decrease of G6P activity (*p* < 0.05, Figure 4A).

#### 2.2.4. Protective Effect on L6 Cells against H_2_O_2_ Stress

L6 myoblast cells were submitted to a pretreatment with Oc extract or Mu extract at a concentration of 50 µg·mL^−1^ for 12 h. Extracts were then discarded by two consecutive washes, and 20 µM of H_2_O_2_ oxidative treatment was applied to induce 50–60% of cells mortality. Under these experimental conditions, the Oc extract pretreatment generated a significant protective effect against H_2_O_2_ treatment (−21% mortality) versus untreated controls (+42% mortality). This benefic effect was equivalent to vitamin C (reference) at 10 µg·mL^−1^ pretreatment. Mu extract pretreatment provoked a marked increase in L6 cells mortality (+22.5% mortality) (*p* < 0.01). Additionally, NCRAE pretreatment demonstrated a moderate but significant protective effect (−15% in mortality) (*p* < 0.05) was drawn (Figure 4B,C).

## 3. Discussion

Building on previous results obtained with a *Chicorium intybus* root extract (NCRAE), the present study designed a one shot strategy to evaluate four in vitro bioactivity tests in order to simplify the determination of the antidiabetic potential of plants. 

We showed that the in vivo antidiabetic property of NCRAE on streptozotocin rats was correlated with a set of four in vitro tests and showed its ability to increase insulin secretion in INS-1 cells, to increase the insulin sensitization of L6 myoblast cells, to protect L6 cells against oxidative H_2_O_2_ stress, and to decrease the glucose-6-phosphatase (G6Pase) activity on rat liver microsomes.

We used two plants, *Ocimum gratissimum* L. leaf extract (Oc) and *Musanga cecropioides* R. Br. ex Tedlie stem bark extract (Mu), well studied for their in vivo antidiabetic effects and known for their hydroxycinnamic contents. The two extracts (Oc and Mu) were analyzed by LC-MS and HPLC to precisely determine their hydroxycinnamic acids contents. Oc contained higher amounts of caffeic acid derivatives than Mu extract, which contained a very high level of apigenin. In contrast, Oc contained a very low amount of apigenin. Both contained chlorogenic acid and caffeic acid but in different proportions. Only Oc contained chicoric acid, which appeared as the major caffeic derivative. The relative content of caffeic derivatives in Mu and Oc was much lower than NCRAE. The variability in the chemical composition of Oc, Mu, and NCRAE extracts was interesting with regard to the perspective of exploring their potential bioactivity profiles.

A DPPH free-radical scavenging experiment highlighted similar potential of all extracts.

Oc extract revealed essential in vitro activities (i) increased insulin secretion from pancreatic β-cells, (ii) increased glucose uptake of muscular L6 cells, (iii) decreased hepatic glucose 6-phosphatase activity, and (iv) clearly reduced H_2_O_2_ oxidative cellular stress. These results tend to demonstrate significant biological activities that may contribute to an improvement of the metabolic disorder associated with metabolic syndrome and T2D. Our results confirm the in vivo hypoglycemic effect of a *Ocimum gratissimum* leaf decoction in normal and streptozotocin-induced diabetic mice [19]. Additionally, as in the precedent work cited, we confirmed the presence of chicoric acid (dicaffeoyl-tartaric acid) in Oc extract.

In comparison with NCRAE, Oc revealed a clear inhibition of the G6Pase activity, which could be correlated with the presence of CGA, known for its molecular interaction with the T1 translocase of the G6Pase complex [32,33].

The cells’ protection against H_2_O_2_ oxidative stress was also in agreement with a recent in vivo study showing that an *Ocimum gratissimum* leaf phenolic extract protected rats against acute inflammation and oxidative stress [18]. Likewise, an aqueous *Ocimum gratissimum* extract revealed antioxidant and cytoprotective activities in the presence of hydrogen peroxide-induced toxicity in human HepG2 cells [34].

Mu extract showed that it (i) inhibited β cells’ insulin secretion, (ii) had no significant effect on muscular glucose uptake, (iii) had no significant effect on G6Pase activity, and (iv) increased mortality of L6 cells under H_2_O_2_ oxidative stress. These results were not in favor of an antidiabetic potential of Mu. They were in contradiction with the in vivo published results. Probably the most controversial of these results was the cellular mortality induced by the extract itself. Apigenin, the most abundant compound in Mu, was reported to reduce the survival of neoplastic cells [35,36]. The inhibition of β cells’ insulin secretion may be explained by the choice of INS-1 cells, which were isolated from a rat insulinoma. Mu extract’s absence of effect on G6Pase activity, despite its CGA content could be attributed to apigenin. Indeed, apigenin was described as a membrane disturber that is able to increase its permeability [37]. The L6 cell mortality could be explained by the presence of protocatechuic acid, known for its selective cytotoxicity on malignant cells with a putative implication of oxidative stress [38]. 

In summary, several conclusions arise from the present study. *Ocimum gratissimum* leaf extract, with its specific composition profile, induced an in vitro antidiabetic response of interest. By contrast, the *Musanga cecropioides* stem bark extract did not manifest satisfactory responses to our in vitro tests. Contrary to NCRAE, we showed that Oc extract revealed the effect of CGA as an inhibitor of hepatic G6Pase activity. These results suggest not only the influence of an extract’s composition profile on biological activity but also the complex interplay between molecules as well as the potential impact of oral absorption when comparing in vivo and in vitro results.

## 4. Materials and Methods

### 4.1. Biological Material and Drugs

Fresh leaves of *Ocimum gratissimum* Linn (herbarium number: 8016/R. Dechamps) and stem bark of *Musanga cecropioides* R. Br. ex Tedlie (herbarium number: 1161/R. Pierlot) were collected in the outskirts of Kinshasa, in district of Kimwenza. The plant material was authenticated by Mr Landu Lukebakio, botanist in the herbarium of the “Institut National des Recherches Agronomiques” (INERA), Faculty of Sciences, University of Kinshasa. A voucher specimen of each species is deposited in the herbarium of the INERA, Faculty of Sciences, University of Kinshasa (Democratic Republic of the Congo). Rat insulinoma-derived INS-1 β cells, were kindly provided by Pr. C.B. Wollheim (Geneva, Switzerland). Rat L6 myoblasts cell-line was purchased from LGC Promochem. Rat L6-Glut4-Myc Myoblasts were obtained from Dr Cabello (France) with the authorization of Dr Klip (USA). Commercial rat liver microsomes were purchased from BD Biosciences (Le Pont de Claix, France). Tolbutamide and cytochalasin B (CCB) were obtained from Sigma-Aldrich (Munich, Germany). Caffeic, chlorogenic, chicoric, and quercetin powders from Sigma-Aldrich (St. Louis, MO, USA) were used to prepare the standard solutions of each compound at 50 µM. 

Oc and Mu extracts were purified using the method previously described [11], with modifications. Briefly, the powder of the part of plant was poured in a cellulose cartridge and placed in a 70% EtOH solution. An extraction of hydrophobic compounds was performed in a separating funnel by a half volume of chloroform before drying. The powder was dissolved in 20% EtOH solution.

### 4.2. Chemical Analysis

#### 4.2.1. LC-MS Analysis

Compounds contained in Oc and Mu extracts were characterized by LC-MS using a Synapt G2-S high-definition mass spectrometry system (Waters Corp., Milford, MA, USA) equipped with electrospray ionization source to characterize the elemental composition of parent and fragment ions according to the procedure previously described [11]. 

#### 4.2.2. HPLC Analysis

Caffeoyl derivatives, essentially chlorogenic acid, caffeic acid, and chicoric acid, were determined and quantified by HPLC coupled with DAD detector. The chromatography system used was an Agilent 1200 series (Agilent, Santa Clara, CA, USA) equipped with a Nova-pak HR C18 250 mm × 4.6 mm × 5 µm column (Waters, Milford, MA, USA). The column thermostat was set to 25 °C, and the samples were injected automatically with an auto-sampler from the same manufacturer and with the volume programmed at 20 µL, corresponding to 20 µg of Oc and Mu extracts. The gradient was set with two phases. Phase A was composed of acetic acid 1%. Phase B was composed of acetonitrile. The program was as follows: time 0 was 100% A; from 0 to 15 min 40% A and 60% B; from 15 to 18 min 20% A and 80% B; from 18 to 25 min 100% A. A standard mix solution containing chicoric acid, chlorogenic acid, caffeic acid, and quercetin, at 10 µM, each was used as a calibrator. The peak areas were determined in order to calculate the concentrations (µM) of each compound and to determine the percentage in the samples. In order to normalize the quantifications of the two extracts, we added in each sample a quercetin solution at a final concentration of 5 µM. The quercetin peak area constituted an external marker.

### 4.3. DPPH Free-Radical Scavenging Capacity

The DPPH (2,2-diphenyl-1-picryl-hydrazyl-hydrate) test was performed as previously described [39] using 100 nmoles of DPPH by assay. The free-radical scavenging activity was defined by the IC_50_, i.e., the quantity of sample needed to obtain 50% of inhibition of the DPPH absorbance was evaluated using quercetin as standard (IC_50_ of 20 µg·mL^−1^). The results were expressed in nmoles of quercetin equivalent (QE) per mg of sample (dry material).

### 4.4. In Vitro Tests 

#### 4.4.1. Insulin Secretion Investigations

The insulin secretion test on rat insulinoma-derived INS-1 β cells was previously described [8]. Briefly, after 5–6 days of cell culture maintained at 37 °C in RPMI-supplemented medium and in a 5% of CO_2_ chamber, cells were washed twice in glucose-free KRB-2% BSA and incubated for 90 min in this medium. Next, cells were washed once in glucose-free KRB-2% BSA and incubated in the same medium added by 5.6 or 11.2 mM glucose. To the 5.6 mM glucose was added or not added Oc and Mu extracts at 50 µg·mL^−1^, with tolbutamide as control (Sigma, Germany) at 100 µM. Insulin released in the medium was determined by the Insulin-Kit HTRFs (Cis-Bio International, Paris, France). Fluorescence intensities were measured on an INFINITE^®^ F500 instrument (TECAN), and results were expressed in percentage of cell contents.

#### 4.4.2. Insulin-Sensitizing Evaluations

The L6 GLUT4-myc line was used to evaluate the level of GLUT4 in the cell membranes by immuno-enzymatic assay, as previously described [40], with modifications. In brief, L6-GLUT4-myc myoblasts were cultured in 96-well plates at 2000 cells/well in DMEM medium supplemented by 10% of FCS [41]. On the day of the experiment, cells were FCS starved in DMEM–0.1% BSA at 37 °C in a 5% CO_2_ chamber during 4 h and washed three times before being incubated one hour in KRB–0.1% BSA, 5 mM glucose, with or without 100 nM insulin, in presence or not of the Oc and Mu extracts at 50 µg·mL^−1^. Cells were washed once with PBS and fixed in 95 °C EtOH during 1 min and washed once with H_2_O. The cell layers were blocked with 3% BSA in PBS at 37 °C for 30 min before being incubated in PBS–0.2% BSA with the primary antibody anti-c-myc 9E10 (1:100) at 37 °C for 4 h. The cells were washed twice with PBS before introducing peroxidase-conjugated rabbit anti-mouse IgG (1:1000). After one hour at room temperature, cells were extensively washed, and 100 µL OPD (o-phenylene-diamine-dihydrochloride) reagent was added to each well. The reaction was stopped by addition of 250 µL of 3 N HCl. The absorbance was measured at 492 nm on a plate-reader, and results were expressed in 492 nm OD × 1000/2000 L6 cells (mean of ten independent assays). L6 cells’ integrity was preserved throughout all experiments, confirming the absence of extract potential toxicity.

#### 4.4.3. Hepatic Glucose 6-Phosphatase (G6Pase) Activity Evaluation on Rat Microsomes

The hepatic G6Pase assay was previously described [9]. Briefly, a hepatic microsome suspension of 10 µL corresponding to 10 µg of total proteins) was incubated for 30 min at 30 °C in a buffer (100 µL) containing 20 mM of glucose 6-phosphate. The effects of Oc and Mu extracts were studied at 50 mg mL^−1^. Positive control was NCRAE [11] at 50 mg·mL^−1^.

The Pi product was determined with malachite green reagent and optical density reading at 660 nm. Results are expressed in pmoles of inorganic phosphate produced during 1 h per 10 µg of total proteins. A Triton X100 hepatic microsome treated was used as a control of microsomes’ integrity. The acidification inhibition served as a negative control.

#### 4.4.4. Protective Evaluation of L6 Cells against H_2_O_2_ Oxidative Stress

As previously described [11], L6-myoblasts cells were seeded at 10^4^ cells/well in 96-well plates and cultured for 4 days in DMEM before changing for a DMEM supplemented with FCS cocktail medium and added with the different extracts at the final concentration of 50 µg·mL^−1^ and cultured 12 h at 37 °C in a 5% CO_2_ chamber. The next day, the cells were washed twice by 200 µL of KRB–0.1% BSA and then placed in the same buffer with or without H_2_O_2_ at 40 µM for three hours to obtain 50–60% of mortality. The cells washed once by PBS were incubated 5 min in 100 µL of Trypan-blue (Sigma-Aldrich) solution 1/3 diluted in PBS. Trypan-blue solution was discarded and the cell layers were visualized with a Stereo Zoom Microscope. The images were treated using ImageJ 1.48v (Image Processing and Analysis in Java: http://imagej.nih.gov/ij/ (accessed on 31 January 2021)) software. The blue color was translated into black spots, and the densities of spots were calculated. The data are expressed as percentage of mortality.

### 4.5. Data Analysis

Statistical analyses were performed using analysis of variance. Data are expressed as mean ± SD. Differences were considered statistically significant at *p* < 0.05 (*), *p* < 0.01 (**), or *p* < 0.001 (***) using the Statgraphics 18^®^ software. 

## 5. Conclusions

In conclusion, the in vitro set of four biological tests, associated with extracts chemical composition analysis, could represent a step toward an alternative method for in vivo studies to use for rapid early screening of putative antidiabetic plant extracts collections. In addition to an obvious interest with regard to ethical animal care issues, a proper cluster of in vitro cellular bioassays focusing on key targets of type 2 diabetes physiopathology may contribute to the anticipation of in vivo pre-clinical therapeutic potential based on proper correlation between in vitro and in vivo results at both the pharmacological and toxicological levels.

In addition, such an optimized combination of in vitro biological tests may provide preliminary insights not only into the ratio of compounds necessary for an extract to produce significant bioactivity but also into the potential deleterious effects at cellular levels; as seen with Mu. Finally, they could provide clues for bioguidance of the extraction process to adequately design plant extracts of therapeutic interest with a proper health benefit/risk ratio.

Further investigations are need to be conducted to integrate the influence of absorption on bioactivity and to confirm and consolidate our data on a larger range of plants.

## Figures and Tables

**Table 1:** Peaks are listed in the order of elution, with their names, molecular formula before ionization, retention times (RT), precursor ions, and fragmentation data of hydroxycinnamoylquinic acids. TR (min): retention time in minutes. Mol. form.: molecular formula. Theo. mass: theoretical monoisotopic mass of precursor ion [M − H]^−^. Δppm: mass tolerance expressed in parts per million.

UV λ_max_ (nm)		Retention Time		Molecular Formula before Ionization	Monoisotopic Ratio *m*/*z*		Mass Deviation			
	Number		Tentative Identification		Theoretical	Observed	Δ (ppm)	Major Fragments	Extract	
(nm)		(min)			[M − H]^−^	[M − H]^−^				
260, 294	P1	1.30	3,4-Dihydroxybenzoic acid (protocatechuic acid)	C_7_H_6_O_4_	153,0188	153,0185	2.0	109	Ocimum	Musanga
256	P2	1.90	4-Hydroxybenzoic acid	C_7_H_6_O_3_	137,0239	137,0235	2.9		Ocimum	
330	P3	2.13	2,5-Dihydroxybenzoic acid (gentisic acid)	C_7_H_6_O_4_	153,0188	153,0187	0.7	109	Ocimum	Musanga
306	P4	2.25	2,3-Dihydroxybenzoic acid	C_7_H_6_O_4_	153,0188	153,0189	−0.7	109		Musanga
309	P5	2.30	3,4-Dihydroxybenzaldehyde	C_7_H_6_O_3_	137,0239	137,0237	1.5	109		Musanga
252, 306sh, 328	P6	2.42	Luteolin-6-C-hexosyl-7-O-hexoside	C_27_H_30_O_16_	609,1456	609,1452	0.7	447, 357, 329, 327, 299	Ocimum	
251, 267sh, 287	P7	2.45	Vanillic acid hexoside	C_14_H_18_O_9_	329,0873	329,0875	−0.6	167, 152, 123, 108		Musanga
252, 267sh, 287	P8	2.54	Vanillic acid = 4-Hydroxy-3-methoxybenzoic acid	C_8_H_8_O_4_	167,0344	167,0341	1.8	108	Ocimum	
230, 301	P9	2.65	Dihydroxybenzoic acid hexoside	C_13_H_16_O_9_	315,0716	315,0718	−0.6	153, 109, 108		Musanga
246, 310sh, 324	P10	2.80	5-O-Caffeoylquinic acid	C_16_H_18_O_9_	353,0878	353,0877	0.3	191, 85	Ocimum	Musanga
302, 348	P11	3.40	Esculetin 6-O-glucoside	C_15_H_16_O_9_	339,0716	339,0719	-0,9	177	Ocimum	
234, 272sh, 334	P12	5.34	Vicenin-2 (Apigenin 6,8-di-C-glucoside)	C_27_H_30_O_15_	593,1506	593,1502	0.7	431, 269, 235, 209, 153, 135	Ocimum	Musanga
232, 268sh, 335	P13	5.87	Apigenin-C-hexosyl-C-hexoside	C_27_H_30_O_15_	593,1506	593,1509	−0.5	473, 383, 353		Musanga
232, 270sh, 338	P14	5.77	Apigenin-C-pentosyl-C-hexoside	C_26_H_28_O_14_	563,1401	563,1404	−0.5	545, 503, 473, 443, 431, 383	Ocimum	
232, 270sh, 316	P15	5.95	Apigenin-C-hexosyl-C-hexoside	C_27_H_30_O_15_	593,1506	593,1504	0.3	473, 383, 353		Musanga
269, 286sh, 337	P16	6.09	Apigenin-8C-hexoside	C_21_H_20_O_10_	431,0978	431,0975	0,7	341, 311, 283	Ocimum	
232, 270sh, 344	P17	6.13	Luteolin-6-C-Xylosyl-8-C-Glucoside	C_26_H_28_O_15_	579,1350	579,1353	−0,5	519, 489, 459, 429, 399, 369, 401		Musanga
232, 269sh, 336	P18	6.27	Apigenin-C-pentosyl-C-hexosyl	C_26_H_28_O_14_	563,1401	563,1407	−1.1	503, 473, 443, 383, 293		Musanga
269, 286sh, 334	P19	6.60	Apigenin-C-hexose-C-xylose p-coumaroyl-ester	C_35_H_34_O_16_	709,1769	709,1766	0.4	563, 545, 420		Musanga
232, 270sh, 337	P20	6.53	Apigenin-C-pentosyl-C-hexosyl	C_26_H_28_O_14_	563,1401	563,1402	−0.2	545, 503, 473, 443, 383		Musanga
269, 286sh, 337	P21	6.76	Apigenin-8C-glucoside (vitexin)	C_21_H_20_O_10_	431,0978	431,0973	1.2	341, 311, 283		Musanga
269, 285sh, 336	P22	6.94	Apigenin-6C-glucoside (isovitexin)	C_21_H_20_O_10_	431,0978	431,0980	−0.5	413, 341, 311, 283		Musanga
241, 310sh, 327	P23	5.87	*trans*-Caftaric acid (2-O-caffeoyl-L-tartaric acid)	C_14_H_14_O_9_	311,0403	311,0401	0.6	179, 149	Ocimum	
232, 272sh, 338	P24	8.70	Apigenin 8- C-rhamnoside-6-hexoside	C_27_H_30_O_14_	577,1557	577,1553	0.7	503, 473, 383, 353		Musanga
262, 277	P25	9.90	Feruloyl tartaric acid isomer 1	C_14_H_14_O_9_	325,0560	325,0555	1.5	193, 178, 149, 134	trace	Musanga
261, 276	P26	10.07	Feruloyl tartaric acid isomer 2	C_14_H_14_O_9_	325,0560	325,0562	−0.6	193, 149, 134	trace	Musanga
261, 275	P27	10.20	Feruloyl tartaric acid isomer 3	C_14_H_14_O_9_	325,0560	325,0559	0.3	193, 149	Ocimum	Musanga
229, 302sh, 325	P28	22.25	3,5-di-O-Caffeoylquinic acid	C_25_H_24_O_12_	515,1195	515,1198	−0.6	353, 191, 179, 173, 175	Ocimum	
241, 297sh, 327	P29	26.15	*trans*-Caffeic acid	C_9_H_8_O_8_	179,0344	179,034	2.2	135, 107	trace	trace
241, 305sh, 327	P30	29.02	Chicoric acid (2,3-dicaffeoyl-L-tartaric acid)	C_22_H_18_O_12_	473,0720	473,0717	0.6	311, 293, 179, 149, 135	Ocimum	Musanga
242, 300sh, 330	P31	35.85	Rosmarinic acid	C_18_H_16_O_8_	359,0767	359,0763	1.1	161, 197, 179	Ocimum	
299, 325	P32	37.10	1,3,5-tri-O-Caffeoylquinic acid	C_34_H_30_O_15_	677,1506	677,1509	−0.4	615, 515, 453, 35, 191, 179, 161	trace	trace

## Data Availability

All data are included on this paper.

## Sample Availability

Samples and compounds are available from the authors.
